# Room Temperature Engineering Crystal Facet of Cu_2_O for Photocatalytic Degradation of Methyl Orange

**DOI:** 10.3390/nano12101697

**Published:** 2022-05-16

**Authors:** Jiwen Li, Meizi He, Jiankun Yan, Jiahui Liu, Jiaxin Zhang, Jingjun Ma

**Affiliations:** 1 College of Science and Technology, Hebei Agricultural University, Huanghua 061100, China; hemeizi21@mails.ucas.ac.cn (M.H.); jkyan007@foxmail.com (J.Y.); jiahuiliu97@163.com (J.L.); zhangjx0926@stumail.ysu.edu.cn (J.Z.); 2 Hefei National Research for Physical Sciences at the Microscale, CAS Center for Excellence in Nanoscience, University of Science and Technology of China, Hefei 230026, China; 3 State Key Laboratory of Coal Conversion, Institute of Coal Chemistry, Chinese Academy of Sciences, Taiyuan 030001, China; 4 College of Environmental and Chemical Engineering, Yanshan University, Qinhuangdao 066004, China

**Keywords:** photocatalysis, Cu_2_O, morphology, adsorption energy, engineering crystal facet

## Abstract

Cuprous oxide (Cu_2_O) has received enormous interest for photocatalysis owing to its narrow band gap of 2.17 eV, which is beneficial for visible-light absorption. In this work, we succeeded in synthesizing Cu_2_O nanocrystals with two morphologies, cube and sphere, at room temperature via a simple wet-chemistry strategy. The morphologies of Cu_2_O change from cube to sphere when adding PVP from 0 g to 4 g and the mainly exposed crystal faces of cubic and spherical Cu_2_O are (100) and (111), respectively. The photocatalytic properties of the as-prepared Cu_2_O were evaluated by the photocatalytic degradation of methyl orange (MO). Cubic Cu_2_O(100) showed excellent photocatalytic activity. After the optical and photoelectric properties were investigated, we found that cubic Cu_2_O(100) has better photoelectric separation efficiency than spherical Cu_2_O(111). Finally, the possible mechanism was proposed for cubic Cu_2_O(100) degrading MO under visible light.

## 1. Introduction

Photocatalysts have received increasing attention because they would bring a broad range of prospects in environment purifying and energy conversion by using solar energy [1,2,3,4]. Therefore, enormous efforts have been made to develop highly efficient photocatalysts and various semiconductor photocatalysts have been obtained in the past few decades. However, some drawbacks have seriously hindered the practical utilization of most photocatalysts, such as the poor light absorption, high recombination of electron–hole pairs, poor stability and high material cost [5,6]. For example, TiO_2_ has been widely used due to its low cost and good stability. Unfortunately, it only photo-responses with ultraviolet (UV) light which accounts for about 4% of solar light [7,8,9]. Ag_3_PO_4_ semiconductor shows excellent performances both in water photooxidation and organic dye photodegradation but is still far from satisfactory for practical applications due to its expensiveness and photocorrosion [10,11]. Therefore, it is a challenge to develop highly efficient photocatalysts with advantages of being visible-light-sensitive and having rapid separation and transfer of photogenerated carriers, high stability and low cost.

Recently, cuprous oxide (Cu_2_O) has attracted tremendous attention due to the fact that it is a p-type narrow-band-gap (2.0–2.2 eV) semiconductor, which is beneficial for visible light absorption. It is also environmentally friendly and low-cost, so it is widely used in energy transformation and environment purification [12,13,14,15]. To date, with the development of the synthesis technique, various shapes and sizes of Cu_2_O’s nanostructure have been fabricated, for instance, Cu_2_O nanowires [16,17,18], nanocubes [15,19,20], polyhedral [21] and nanospheres [22]. For example, Zhang et al. [19] successfully synthesized a series of architectures of Cu_2_O with PVP as a capping agent and used them for photocatalytic degradation of MO. Huang et al. [23] prepared cubic and octahedral Cu_2_O nanocubes with and without PVP, respectively. Liang et al. [24] obtained various Cu_2_O single crystals through a sample method with oleic acid as capping agent and D-(+)-glucose as reduction agent at 100 °C in the water phase. Zeng et al. [25] used copper(II) acetate as a precursor, hexa-decylamine as the surfactant and undecane as a reduction agent to prepare Cu_2_O nanocrystals. However, most of the preparation strategies for Cu_2_O require complicated processes. So, it is important to develop new synthesis methods in relatively low temperatures for Cu_2_O. Up to now, there have been great advances in the synthesis of nanomaterials. For instance, Mojtaba Bagherzadeh and co-workers fabricated Cu_2_O nanocrystals using CuCl_2_ as the raw material and operated in a water bath at 40 °C [26]. Fang et al. [27] obtained Cu_2_O nanocubes by a simple route at room temperature which were used as a template to prepare CuS@Co(OH)_2_ nanoboxes. In addition, they also used Cu_2_O nanocubes obtained at room temperature as a template to prepare a hollow nanomaterial [28]. Therefore, developing a simple synthesis strategy to obtain different morphologies in Cu_2_O nanocrystals with excellent properties for photocatalysis at room temperature is an important challenge, and there are few studies on the effect of crystal plane and morphology on catalytic performance.

In this work, a simple strategy was developed to synthesize Cu_2_O nanocrystals by using CuSO_4_∙5H_2_O as a precursor, PVP as a capping agent and ascorbic acid (AA) as a reduction agent at room temperature. The shapes of Cu_2_O changed from cubic to spherical when adding the amount of PVP from 0 g to 4 g, and the mainly exposed crystal faces of cube and spherical Cu_2_O were (100) and (111), respectively. The as-prepared samples were used in the photocatalytic degradation of dye to investigate the relationship between shape and activity. According to research findings, cubic Cu_2_O(100) showed excellent photocatalytic activity. Subsequently, optical and photoelectric properties of the as-prepared Cu_2_O nanocrystals were investigated, and the possible mechanism was proposed. This work is beneficial to develop new synthesis methods of Cu_2_O. 

## 2. Experimental Section

### 2.1. Materials

Cupric sulfate (CuSO_4_∙5H_2_O) (≥99%) was purchased from Tianjin Sheng’ao Chemical Reagent Co. Ltd (Tianjin, China). Trisodium citrate (C_6_H_5_Na_3_O_7_∙2H_2_O) (≥99%) was purchased from Tianjin Tianda Chemical Reagent Factory (Tianjin, China). Polyvinylpyrrolidone (PVP)(1000~1,300,000) and ascorbic acid (AA) (≥99.7%) were purchased from Tianjin Kemeiou Chemical Reagent Co. Ltd (Tianjin, China). Sodium hydroxide (NaOH) (≥96%) was purchased from Tianjin Fengchuan Chemical Reagent Technology Co. Ltd (Tianjin, China). Isopropanol (IPA) was purchased from Hangzhou Shuanglin Chemical Reagent Co., Ltd (Hangzhou, China). and p-benzoquinone (BQ) was purchased from Chengdu Aikeda Chemical Reagent Co., Ltd (Chengdu, China). Ethylenediaminetetraacetic acid (EDTA) was purchased from Tianjin Damao Chemical Reagent Factory (Tianjin, China). The reagents used are all analytically pure. All reagents are analytical grade without further purification. Deionized (DI) water in the experiment was obtained from local sources. 

### 2.2. Sample Preparation

#### 2.2.1. Synthesis of Cubic Cu_2_O (100) Nanocrystals

Synthesis of cubic Cu_2_O was similar with previously reported [27]. Typically, CuSO_4_∙5H_2_O (1.5 mmol) and C_6_H_5_Na_3_O_7_∙2H_2_O (0.5 mmol) were added into deionized water (80 mL) and vigorously stirring for 1 h until the powder dissolved completely. Then, 20 mL NaOH solution (1.25 M) was added in to the above solution dropwise under vigorous stirring, and the solution turned from light blue to dark blue gradually, which indicated the formation of Cu(OH)_2_ precipitate [28]. Afterward, 50 mL AA (0.03 M) was added to the suspension dropwise under stirring. The color of solution turned into dark brown and finally became brick red. Subsequently, the above mixture was stirred evenly for 1 h for nanocrystal growth, and then before standing for 2 h. The whole operation was carried out at room temperature. Finally, the resulting precipitates were collected by centrifugation, and washed again using the same volume ratio mixture of water and absolute ethanol to remove residual chemicals three times. The brick-red solids were obtained by drying in vacuum at 50 °C for all of the night. The obtained Cu_2_O nanocrystals were named c-Cu_2_O(100). 

#### 2.2.2. Synthesis of Spherical Cu_2_O(111)

Spherical Cu_2_O-4 (111) was prepared by the same synthesis route as for cubic Cu_2_O (100), but a certain amount of PVP to the aqueous solution of CuSO_4_∙5H_2_O and C_6_H_5_Na_3_O_7_∙2H_2_O was added. The amount of PVP was adjusted from 0.5 g, 1 g, 2 g, 3 g, to 4 g, and the corresponding obtained Cu_2_O nanocrystals were labeled as Cu_2_O-0.5, Cu_2_O-1, Cu_2_O-2, Cu_2_O-3, and Cu_2_O-4 (111), respectively.

### 2.3. Characterization

Powder X-ray diffraction patterns (XRD) were carried out at a Bruker D8 Advance powder X-ray diffractometer (Bruker, Karlsruhe, Germany) using Cu Kα radiation (40 kV, 40 mA) with scanning range from 20° to 80° at a scan rate of 0.02° per second. The morphology of samples was determined by the scanning electron microscopy (SEM) measurements on a FEI QUANTA Q400 scanning electron microscope (FEI, Pittsburgh, PA, USA). The UV-visible diffuse reflectance spectra (DRS) of powder samples were recorded on a Hitachi UH4150 spectrophotometer (Hitachi, S 4800, Tokyo, Japan) with BaSO_4_ as a reflection standard in the range of 300–800 nm. Transmission electron microscopy (TEM) images and the corresponding selected area electron diffraction (SAED) patterns were conducted by an FEI Tecnai G2 F20 field emission TEM (Nicolet, Green Bay, WI, USA). X-ray photoelectron spectroscopy (XPS) analysis was probed on a Thermo Scientific ESCA Lab 250Xi spectrometer (Thermo Fisher Scientific, West Sussex, UK) with a monochromatic Al Kα (hν = 1486.6 eV) as the X-ray source, and C 1s peak at 284.4 eV was used to calibrate the binding energy before spectra deconvolution. Fourier-transform infrared (FTIR) spectra were carried out using a Nicolet iS5 FTIR spectrometer (Nicolet, Green Bay, WI, USA), using the standard KBr wafer technique. Photoluminescence (PL) and time-resolved photoluminescence decay (TRPL) spectra of samples were recorded on HITACHI F-7000 fluorescence spectrophotometer (Hitachi, Tokyo, Japan) and FLSP920 fluorescence lifetime spectrophotometer (Edinburgh Instruments, Edinburgh, UK), respectively.

### 2.4. Photoelectrochemical Measurements

The photoelectrochemical (PEC) tests were carried out on a CHI-760E electrochemical workstation (Chenhua Instrument, Shanghai, China) in a conventional three-electrode cell. Pt plate and Ag/AgCl were used as counter and reference electrodes, respectively. The working electrode was obtained by dip-coating catalyst slurry on FTO glass. FTO glass was cleaned by acetone and ethanol for 20 min each, then washed with DI water. Catalyst suspension was prepared by dispersing c-Cu_2_O(100) and Cu_2_O-4(111) powder (5 mg) in absolute ethyl alcohol (5 mL) by sonication for 30 min, then 0.15 mL slurry was dip-coated on conductive surface of FTO glass, and the electrode area was controlled at 1 cm^2^. Finally, the working electrode was dried at room temperature in the air for one night. During photoelectrochemical testing, 0.1 M Na_2_SO_4_ was used as electrolyte and a 300 W Xe lamp with UV filter was used as incident light.

### 2.5. Photocatalytic Performance Test

The photocatalytic activity of the obtained Cu_2_O nanocrystals was evaluated via photocatalytic decomposition of model dye MO aqueous solution under visible light irradiation. The detailed steps were as follows: first, 50 mg of the as-prepared samples was dispersed into MO solution (100 mL, 0.05 mM) under stirring for 0.5 h in a jacketed beaker as reactor with a circulating water system running through the jacket to keep the temperature constant during the reaction progress. Second, to ensure absorption–desorption equilibrium between catalysts and MO, the mixture was magnetically stirred in dark for 30 min before irradiation. Visible-light source was provided by 300 W Xe lamp (Beijing prinsess Technology Co., Ltd., Beijing, China) with a UV cutoff filter (λ > 420 nm), and the distance of liquid level and light source was 15 cm. The experimental device is shown in Figure S1. Third, 3 mL suspension was sampled and centrifuged (4000 r/min, 4 min) to remove the particles at certain time intervals after irradiation with visible light. The concentration of MO was detected by TU-1810 UV-vis spectrophotometer at 464 nm.

The kinetic curves of photocatalytic MO degradation can be expressed and the rate constants can be further calculated by the pseudo-first-order Equation (1) [29]. The degradation rate can be used to investigate photocatalytic efficiency at a given time, the degradation rate (η) can be calculated with the following Equation (2):

−In (C_t_/C_0_) = Kt (1)

η (%) = (C_0_ − C_t_)/C_0_ × 100% (2)

where K (min^−1^) is the rate constant, C_0_ (mg·L^−1^) is the initial concentration of MO and C_t_ (mg·L^−1^) is the concentration of MO at time t (min), respectively.

### 2.6. Trapping Experiment of Active Species

The electron spin resonance (ESR) measurements were performed with a JES FA200 (JEOL, Tokyo, Japan) spectrometer, with spin-trap reagent 5, 5-dimethyl-1-pyrroline N-oxide (DMPO) in water for hydroxyl radical (·OH) and methanol for superoxide radical (O_2_·^−^) at room temperature. To investigate the main reactive active species responsible for the degradation of MO over Cu_2_O nanocrystals, the radical species scavengers experiment was implemented. Some quenchers were used to scavenge the relevant reactive active species. BQ, IPA, EDTA were employed to scavenge O_2_·^−^, ·OH, and hole, respectively [30,31,32]. The concentration of the above scavenger reagents in mixture solutions was 0.1 mM to BQ, 2 mM to IPA, and 2 mM to EDTA.

## 3. Results and Discussion

### 3.1. Synthesis

**Scheme 1:**
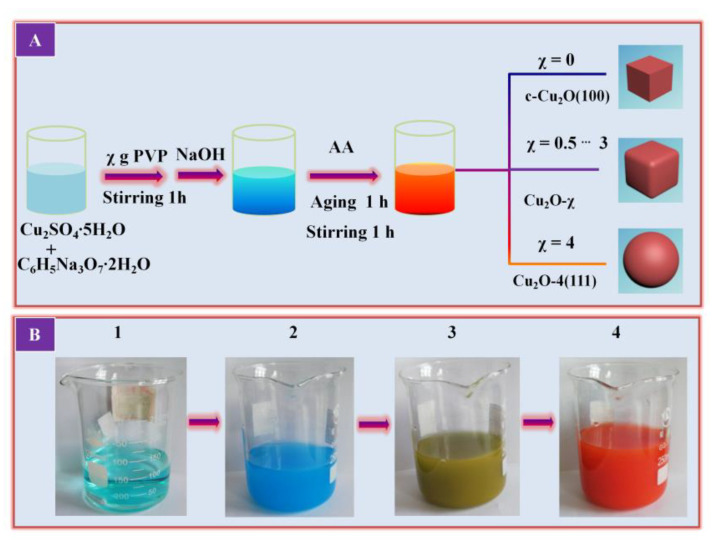
(**A**) Schematic illustration for the preparation of Cu_2_O growth process, (**B**) the color change of solution during preparation progress of Cu_2_O nanocrystals. In Figure (**A**), **χ** represents the amount of PVP added. The numbers in Figure (**B**) represent the different stages of preparation of cuprous oxide.

The Cu_2_O nanocrystals used in this work were synthesized via a modified route [19,22,27]. The typical preparation is depicted in Scheme 1A. First, with CuSO_4_∙5H_2_O as copper source and C_6_H_5_Na_3_O_7_∙2H_2_O sufficiently dissolving in DI, the solution displayed a pale blue color. Second, a different amount of PVP was added into the above solution and it was kept stirring for a long time until the PVP dissolved completely. Third, NaOH solution was added to the above solution dropwise under vigorous stirring, and the solution turned from light blue to dark blue gradually. Finally, reductant AA was added to the suspension dropwise under stirring. The color of the solution turned into dark brown and became brick red finally. This color indicated Cu_2_O nanocrystals were successfully fabricated. It is worth noting that the second step is an important step for the controlling of Cu_2_O nanocrystals. The final products exhibited a cubic shape if there was no PVP added into the mixture in the second step. The shapes of the Cu_2_O nanocrystals were changed from cubic to spherical when the amount of PVP added changed from 0 g to 4 g.

The possible reaction of Cu_2_O nanocrystals based on the above fabrication process was proposed. CuSO_4_∙5H_2_O solid was dissolved in DI to form Cu^2+^, and the solution was labeled as solution (1). The light blue is the inherent color of CuSO_4_ aqueous solutions as shown in the first picture in Scheme 1B. Subsequently, NaOH aqueous solution was added to the solution (1) dropwise; the Cu^2+^ reacted with OH^-^ and formed Cu(OH)_2_ precipitate rapidly. The suspension liquid was labeled as suspension (2). The dark blue color of the suspension (2), as shown in the second picture in Scheme 1B, was the inherent color of the Cu(OH)_2_ precipitate. After AA was added, the AA firstly reacted with the remaining OH^−^ from NaOH. When the added amount of AA increased, the pH value of the mixture solution changed from alkaline to neutral. If continuing to add AA in the above mixture, the pH value of the mixture will tend to be acidic, and AA has a reducing capacity in this case, and Cu(Ⅱ) in Cu(OH)_2_ was reduced to Cu_2_O; the color of the third picture in Scheme 1B (mixture (3)) verified the formation of Cu_2_O [33]. When adding enough AA in to mixture (3), the Cu(Ⅱ) was reduced to Cu_2_O completely, and the brick-red color of the fourth picture in Scheme 1B was the inherent color of Cu_2_O. The possible occurred chemical reaction equations of solution (1), (2) and mixture (3) were also proposed as the following [34]:

(1) CuSO_4_∙5H_2_O aqueous solutions CuSO_4_→Cu^2+^ + SO_4_^2−^ (light blue);
(2) Adding NaOH aqueous solution in to (1) Cu^2+^ + 2OH^−^→Cu(OH)_2_↓ (dark blue);
(3) Adding AA aqueous solution in to (2)

2Cu(OH)_2_↓ + 2AA(e−)→Cu_2_O↓ + H_2_O + 2OH^−^ (brick red).

### 3.2. Crystal Structures Information

**Figure 1:**
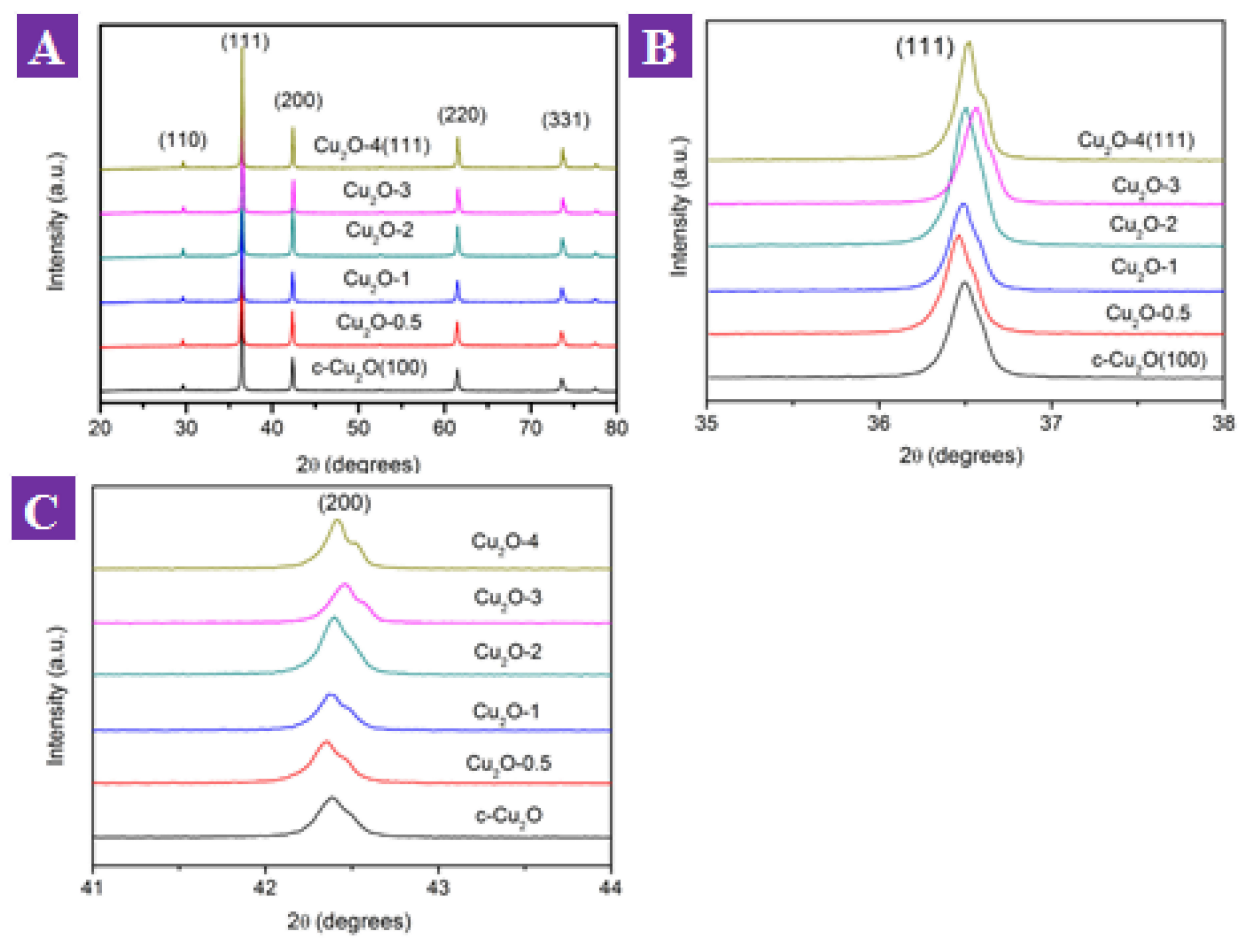
XRD profiles of the as-prepared samples. Enlarged view of the sample (**A**) at (**B**) (111) and (**C**) (200) crystal planes.

The structure information and phase purities of as-prepared samples were investigated by X-ray diffraction (XRD). As shown in Figure 1A, it is the unique diffraction pattern of the Cu_2_O nanocrystals, namely the peaks at 2θ of 29.3°, 36.2°, 42.1°, 61.3°, and 73.4° belonging to the (110), (111), (200), (220), and (311) planes of Cu_2_O, respectively, which correspond to the cubic bulk of Cu_2_O (JC-PDS file No. 78-2076) [26,35]. Beyond that, other peaks were negligible, which implied that as-prepared Cu_2_O nanocrystals are relatively pure. The diffraction patterns of Figure 1B,C are the images of (111) and (200) planes of Cu_2_O samples, respectively. It can be seen that there is a very small peak to the right of the main peak here, which gradually becomes larger as the exposed surface of Cu_2_O changes, probably due to the influence of morphology, size or exposed crystal surface.

### 3.3. Morphology Measure and Discussion

**Figure 2:**
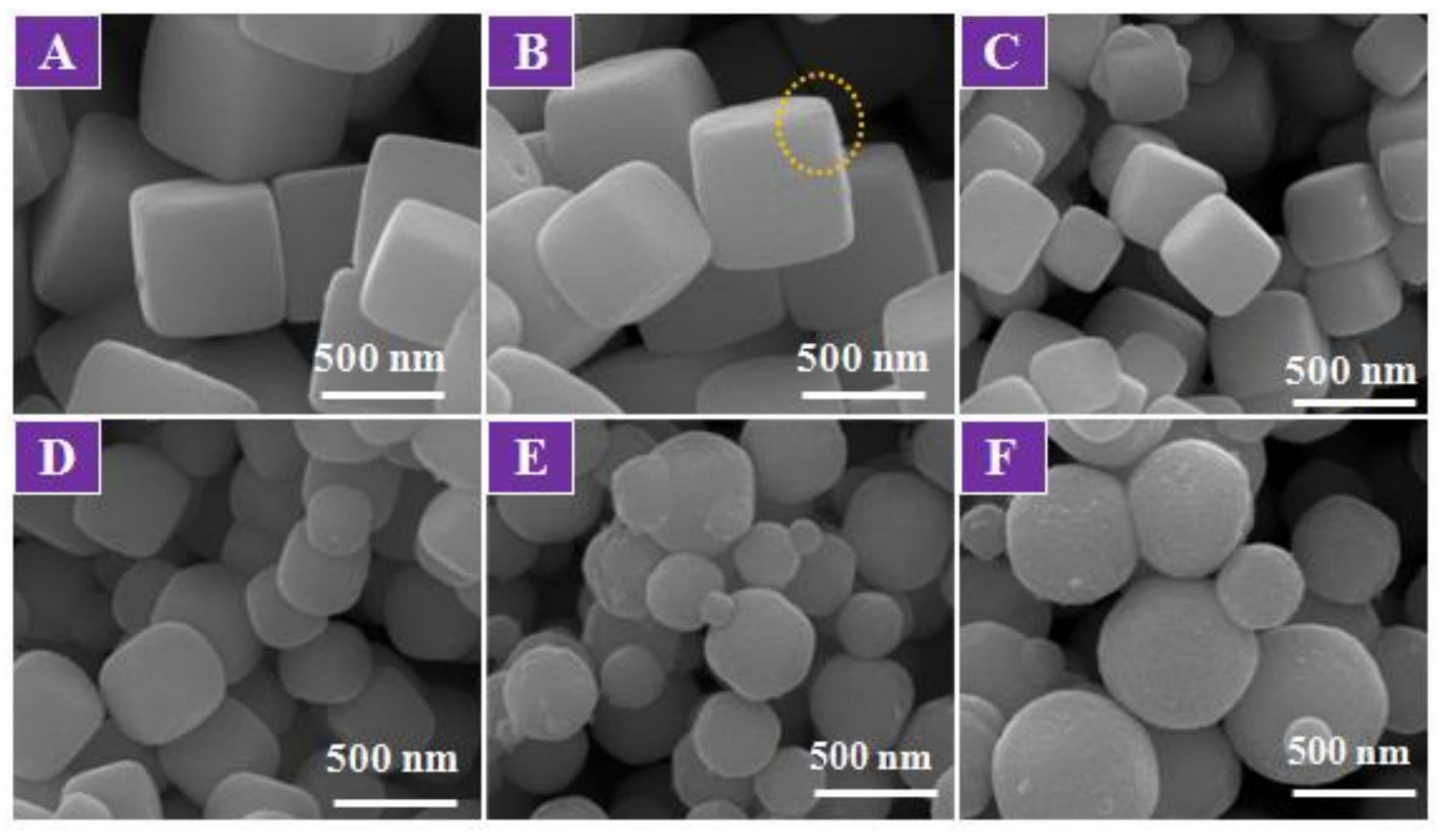
SEM images of the as-prepared samples: (**A**) c-Cu_2_O(100), (**B**) Cu_2_O-0.5, (**C**) Cu_2_O-1, (**D**) Cu_2_O-2, (**E**) Cu_2_O-3 and (**F**) Cu_2_O-4(111).

The geometric shape and microstructures of as-prepared samples have been analyzed by scanning electron microscopy (SEM). As shown in Figure 2A, in the absence of PVP, the Cu_2_O nanoparticles possess cubic morphology and a smooth surface, which is corresponding to the previous reports [19,27,34,36]. The length of the c-Cu_2_O(100) cube was about 500 nm. When adding different amounts of PVP, the corners of cubic Cu_2_O disappeared and became a round arc structure as shown in the yellow circle of Figure 2B, and the sizes of the nanoparticles barely changed. The round arc of the corners of c-Cu_2_O(100) gradually enlarged after continuing addition of PVP to 1 g as shown in Figure 2C; the diameter of Cu_2_O-1 was about 400 nm. When the amount of PVP increased to 2 g, the morphology of the nanoparticles was nearly spherical, with uneven size from 400 to 100 nm (Figure 2D). When PVP was increased to 3 g or 4 g, the Cu_2_O-3 and Cu_2_O-4(111) displayed spherical structures, where Cu_2_O-4(111) had a more complete sphere shape Cu_2_O-3, as shown in Figure 2E,F. 

**Figure 3:**
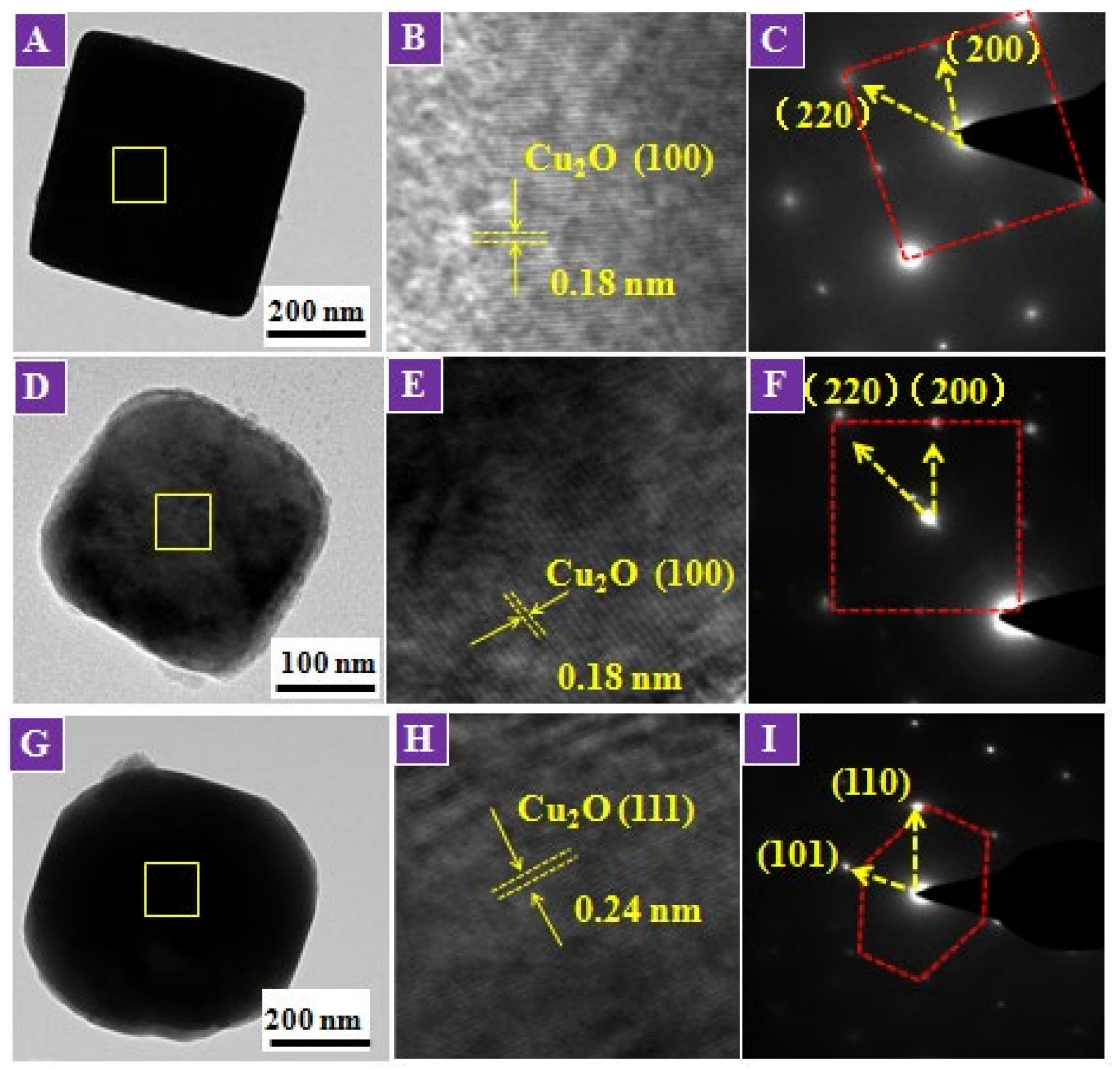
The detailed crystallographic features of the Cu_2_O nanocrystals. (**A**,**D**,**G**) the bright TEM images, (**B**,**E**,**H**) high-resolution TEM (HRTEM) image, (**C**,**F**,**I**) the corresponding SAED of c-Cu_2_O(100), Cu_2_O-1 and Cu_2_O-4(111), respectively. The yellow square border in (**A**,**D**,**G**) represents the selected area on the sample to be enlarged for HRTEM.

To further investigate the crystallographic features, the TEM, HRTEM and the corresponding SAED analysis were performed. As shown in Figure S2 and Figure 3, the changing trend in the shape and crystal planes for Cu_2_O nanocrystals can be clearly observed. Take Figure 3A for an example; c-Cu_2_O(100) exhibited a relatively well-defined cube shape. The high-resolution TEM (HRTEM) image as shown in Figure 3B, which exhibits clear fringes with lattice spacing of 0.18 nm, corresponds to the (100) surfaces of Cu_2_O nanocrystals, and the corresponding SAED pattern also indicated c-Cu_2_O(100) exposes the (100) surfaces (Figure 3C). Figure 3D shows the TEM image of Cu_2_O-1; its corners disappeared and gradually became a round arc compared to c-Cu_2_O(100). When sited on the yellow region, namely the surface of Cu_2_O-1, the results of the HRTEM image (Figure 3E) and SAED pattern (Figure 3F) agree with those of c-Cu_2_O(100), which indicated that Cu_2_O-1 exposes (111) surfaces. As mentioned about the section of Figure 2, the round arc corner gradually enlarged from cube to sphere after continuing addition of PVP from 0 to 4 g. For further understanding of this changing, TEM, HRTEM and the corresponding SAED measurements were performed to Cu_2_O-4(111). As shown in Figure S2C and Figure 3G, Cu_2_O-4(111) possesses spherical morphology and the lattice spacing of about 0.24 nm (Figure 3H), which correspond to the (111) lattice planes of Cu_2_O. Exposed (111) facets were further demonstrated by the corresponding SAED pattern (Figure 3I). These results about Cu_2_O nanocrystals agreed with the previous reports [19,20,37].

**Figure 4:**
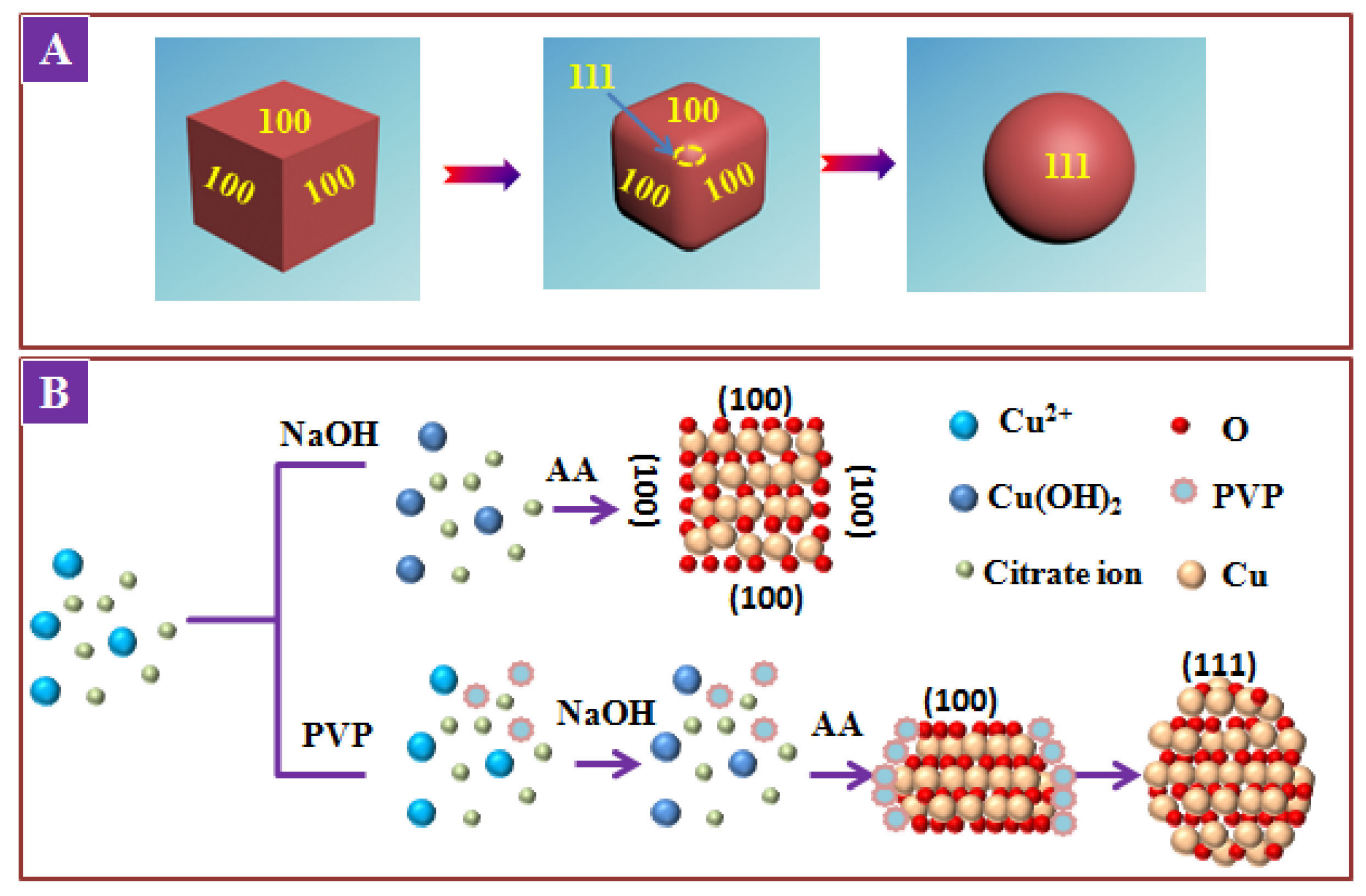
(**A**) the changing of exposing surface and (**B**) the role of PVP during Cu_2_O nanocrystal preparation process.

Based on the above results, the relationship between Cu_2_O nanocrystals’ shape and surfactant PVP has been discussed. As shown in Figure 4A, the three-dimensional models clearly demonstrated the exposed surfaces’ evolution process from (100) to (111) with the shape of Cu_2_O nanocrystals changing from cube to sphere after increasing the amount of PVP, respectively. Therefore, it is not difficult to find that PVP acts as a capping agent, and it can be preferential in adsorption on the {111} planes of the Cu_2_O nanocrystals to protect (111) facets as shown in Figure 4B [19]. Therefore, by adjusting the amount of PVP to control the adsorption kinetic of the surface activities on {111} facets, the area of (111) surfaces enlarge gradually until completely exposing the (111) facets by increasing the amount of PVP to 4 g. According to reports in the literature, sodium citrate was used as a chelating agent to adjust the size of Cu_2_O nanocrystals via nucleation-controlled route [34]. 

### 3.4. Chemical Coordination Analysis

**Figure 5:**
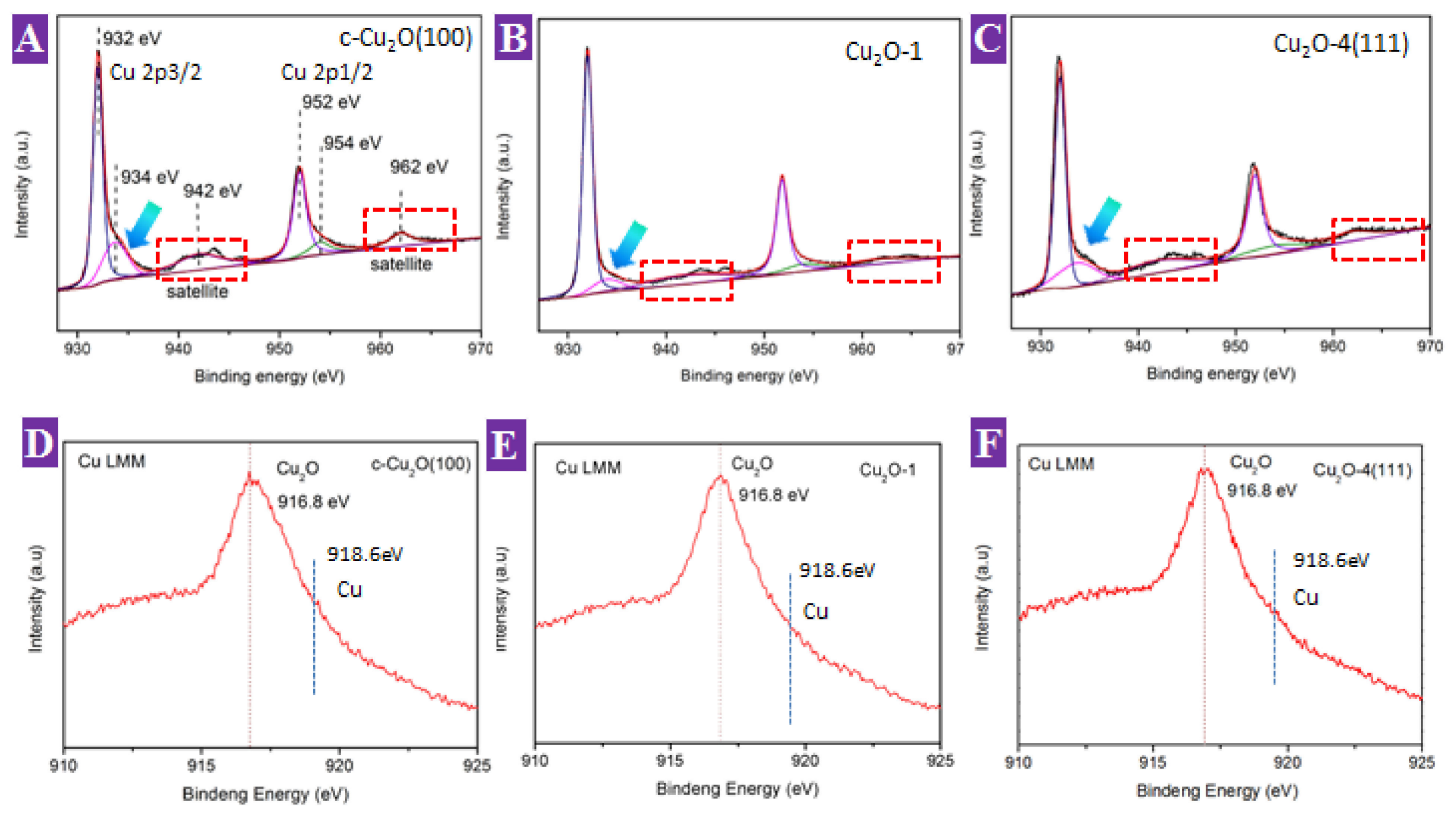
XPS spectra of the as-prepared samples. (**A**–**C**) Cu2p high-resolution spectra of, (**D**–**F**) Cu LMM of c-Cu_2_O(100), Cu_2_O-1 and Cu_2_O-4(111), respectively.

XPS is used to analyze the surface composition and electronic environment of corresponding elements for as-prepared samples. XPS also can be used to further verify the purity of samples. As shown in Figure S3, the peaks of O, Cu and adventitious C elements can be clearly observed, with no other peak emerging, which indicated that the as-prepared Cu_2_O nanocrystals (c-Cu_2_O(100), Cu_2_O-1 and Cu_2_O-4(111)) were highly pure [38]. For further investigating Cu’s chemical state on the surface of samples, the high-resolution XPS spectra of Cu 2p were also examined. As shown in Figure 5A–C, there were two main characteristic peaks corresponding to Cu 2p1/2 and Cu 2p3/2, and the distance between the two main peaks is about 19 eV, which was in agreement with the previous reports [39,40,41]. There are several shake-up satellite peaks emerging at nearby 942 and 952 eV (red dotted box mark), as previously reported [39], approximately 9 eV higher than the value of the Cu 2p3/2 peak; these peaks may be attributed to multiple excitations in copper oxides such as the characteristic peaks of CuO. There is a slight bulge that can be observed to the right of the peaks of Cu 2p3/2, as shown in Figure 5A–C (blue arrow mark), which indicated that there may be Cu^2+^. The peaks at 932 eV (Cu 2p3/2) and 952 eV (Cu 2p1/2) can be assigned to Cu^+^ (originated from Cu_2_O) and the peaks at 934 (Cu 2p3/2) and 954 eV (Cu 2p1/2) corresponding to the state of Cu^2+^ (originated from CuO) [42,43]. The existence of CuO indicates that the surface of c-Cu_2_O(100) is oxidized from Cu_2_O to CuO. The intensity of the peak of Cu^+^ 2p3/2 is much larger than that of the peak of Cu^2+^ 2p3/2, indicating that the dominant component of c-Cu_2_O(100) nanocrystals is Cu_2_O, which is consistent with report [43]. Comparing with Figure 5A–C, there is less oxidation which occurs on the surface of Cu_2_O-1. The successful preparation of Cu_2_O nanocrystals is also verified by the Cu LMM spectrum, as shown in Figure 5D–F. The only peak can be observed at 916.8 eV corresponding to Cu^+^; there is no peak of Cu (at 918.6 eV) emerging. As mentioned previously, the result of XRD indicated that the as-prepared samples only possessed a Cu_2_O phase, which was different from that of XPS because the XPS technique is used to study the surface of a sample, unlike XRD which is for the bulk [40]. These further verified that very little oxidation took place on the surface of c-Cu_2_O(100) and Cu_2_O-4(111). From the O 1s XPS spectrum in Figure S4, we observe that the prepared samples had three peaks at about 530.1 eV, 531.5 eV and 533.0 eV. The peak at 530.1 eV was attributed to the oxygen of Cu_2_O and CuO. The peak at 531.5 eV was assigned to adsorbed oxygen on the surface. The peak at 533.0 eV was associated with the adsorbed water molecules [23].

### 3.5. Optical Properties

**Figure 6:**
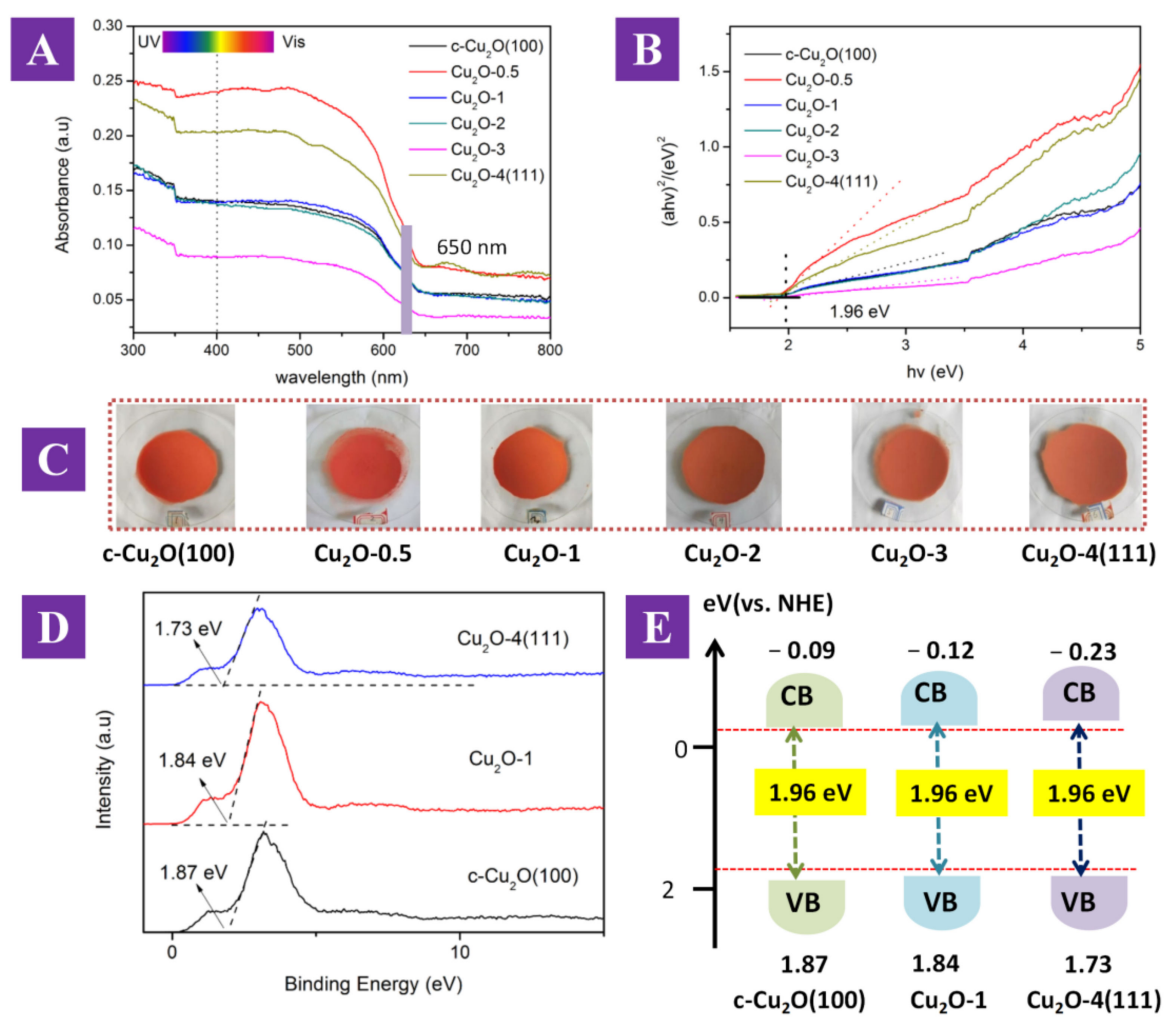
(**A**) UV–vis diffuse reflectance spectra and (**B**) plots of (αhν)^2^ versus energy (hν) of the as-prepared samples. c-Cu_2_O(100), Cu_2_O-0.5, Cu_2_O-1, Cu_2_O-2, Cu_2_O-3 and Cu_2_O-4(111), respectively. (**C**) The corresponding sample images, (**D**) Valence band XPS spectra of the c-Cu_2_O(100), Cu_2_O-1 and Cu_2_O-4(111). (**E**) Schematic of energy band structure.

The UV-vis absorption spectroscopy and XPS valence spectra were performed to investigate the optical absorption property and electronic structure of the as-prepared samples [44,45]. As shown in Figure 6A, the light absorption edges of Cu_2_O nanocrystals were all around 650 nm (purple column mark), which indicated that Cu_2_O nanocrystals possess good optical absorption property, which can be excited by visible light. However, there was no relationship between light absorption and morphology in this work, namely, Cu_2_O-0.5 showed the strongest absorption but Cu_2_O-3 showed the weakest absorption in the visible light region. The band gap value obtained from the square root plots of (αhν)^2^ versus energy (hν) of samples is shown in Figure 6B; all the as-prepared samples have the same band gap of 1.96 eV, which corresponds to the result of UV-vis absorption spectroscopy. The color of the samples also can further verify the light absorption performance as shown in Figure 6C; all the samples have the same color of brick red. For investigating the band position of the as-prepared samples, the valence band XPS spectroscopy was carried out. As presented in Figure 6D, the samples c-Cu_2_O(100), Cu_2_O-1 and Cu_2_O-4(111) displayed the valence band (E_VB_) maximum of 1.87, 1.84 and 1.73 eV, respectively. According to the band gap value in Figure 6B, combined with calculation formula Eg = E_VB_ − E_CB_ (E_CB_ is conduction band) [46,47], the minimum E_CB_ of c-Cu_2_O(100), Cu_2_O-1 and Cu_2_O-4(111) occur at about −0.09, −0.12 and −0.23 eV, respectively (Figure 6D). Based on the above results, it is found that the obtained samples have almost the same light absorption performance except the minor differences in band position.

### 3.6. Photocatalytic Performance Tests

**Figure 7:**
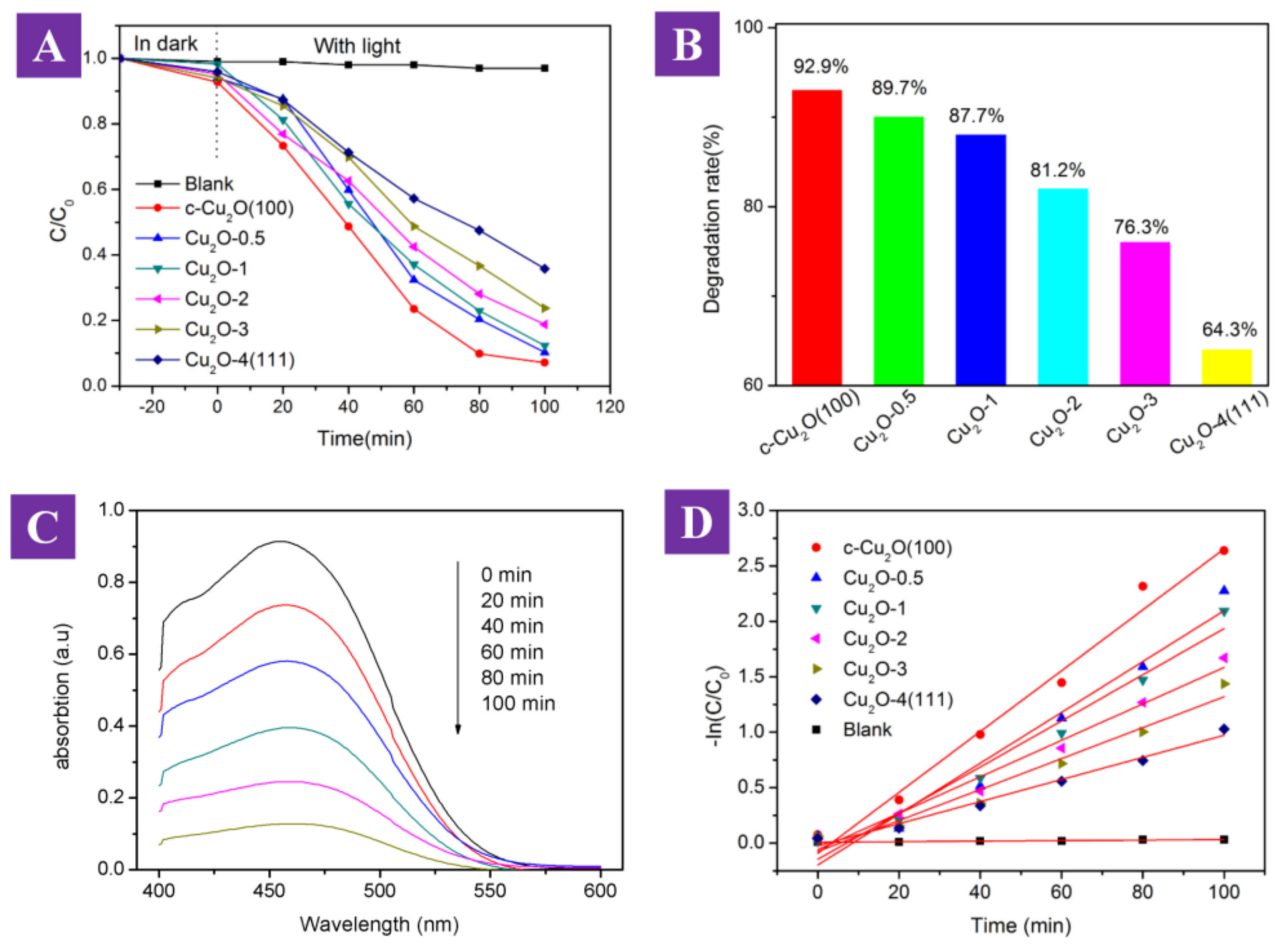
(**A**) Photocatalytic degradation of MO by the as-prepared samples. (**B**) The corresponding MO degradation ration. (**C**) UV-vis absorption spectra of MO with c-Cu_2_O nanocrystals under visible light irradiation. (**D**) Comparison of the corresponding MO degradation kinetics.

The photocatalytic degradation MO test was carried out to evaluate photocatalytic performance of the obtained samples under visible-light irradiation. As shown in Figure 7A, MO was barely adsorbed by the Cu_2_O nanocrystals during the absorption–desorption balance test under in dark. For comparison, a blank experiment also implied this, and the MO concentration remained unchanged over time in the absence of catalysts, indicating that MO is quite stable. When adding Cu_2_O nanocrystals, the concentration of MO decreased with the reaction time under visible-light irradiation. Among them, c-Cu_2_O(100) exhibited the best photocatalytic degradation performance and could completely decompose MO in aqueous solution within 80 min. It is notable that the photocatalytic degradation performance is related to the crystal plane of Cu_2_O nanocrystals; the order of degradation efficiency for obtained samples was: c-Cu_2_O(100) (92.8%) > Cu_2_O-0.5 (89.7%) > Cu_2_O-1 (87.7%) > Cu_2_O-2 (81.2%) > Cu_2_O-3 (76.3%) > Cu_2_O-4(111) (64.3%) as shown in Figure 7B. Figure 7C shows the UV-vis absorption spectra of MO with c-Cu_2_O nanocrystals under visible-light irradiation; the concentration of MO decreased gradually under continuous light irradiation. The degradation of MO could be described by a pseudo-first-order reaction with a modification-simplified Langmuir–Hinshelwood model as aforementioned in Equation (1), as shown in Figure 7D. The corresponding rate constant K of as-prepared samples c-Cu_2_O(100), Cu_2_O-0.5, Cu_2_O-1, Cu_2_O-2, Cu_2_O-3 and Cu_2_O-4(111) are deduced to be 0.027, 0.023, 0.021, 0.016, 0.014 and 0.010, respectively. Based on the above results, it is found that the photocatalytic performance decreases gradually with the shapes of Cu_2_O nanocrystals changing from cube to sphere and the exposed surfaces changing from (100) to (111).

As mentioned in Figure 5, there is little oxidation taking place on the surface of c-Cu_2_O(100) and Cu_2_O-4(111), which would have affected and suppressed the photodegradation performance [43]. As mentioned in Figure 6, the obtained samples have almost the same light absorption performance except for the minor differences in band position. These results indicate that the Cu_2_O nanocrystals may have the same photocatalytic performance. However, the photodegradation efficiency is decreased gradually when the shapes of Cu_2_O nanocrystals change from cube to sphere with the exposed surfaces changing from (100) to (111). It is further verified that the crystal facet may be the key factor for affecting the photocatalytic performance of Cu_2_O nanocrystals. In addition, the structural information for samples was measured by FTIR. As shown in Figure S5, c-Cu_2_O(100), Cu_2_O-1 and Cu_2_O-4(111) all show the same representative peaks at 632 cm^−1^ and 1632 cm^−1^ which are attributed to the Cu–O bond stretching vibration and –OH bending vibration (originating from the surface-adsorbed H_2_O) [19], respectively. The results further corroborate that the obtained samples are Cu_2_O and no PVP exists after centrifugation and washing three times; the result is consistent with that of O 1s XPS spectrum (Figure S4). According to the previous reports [19,21], the as-prepared Cu_2_O nanocrystals may possess a facet-related photocatalysis ability for MO, or edges and corners may have the dominant catalytic activity for Cu_2_O nanocrystals.

**Figure 8:**
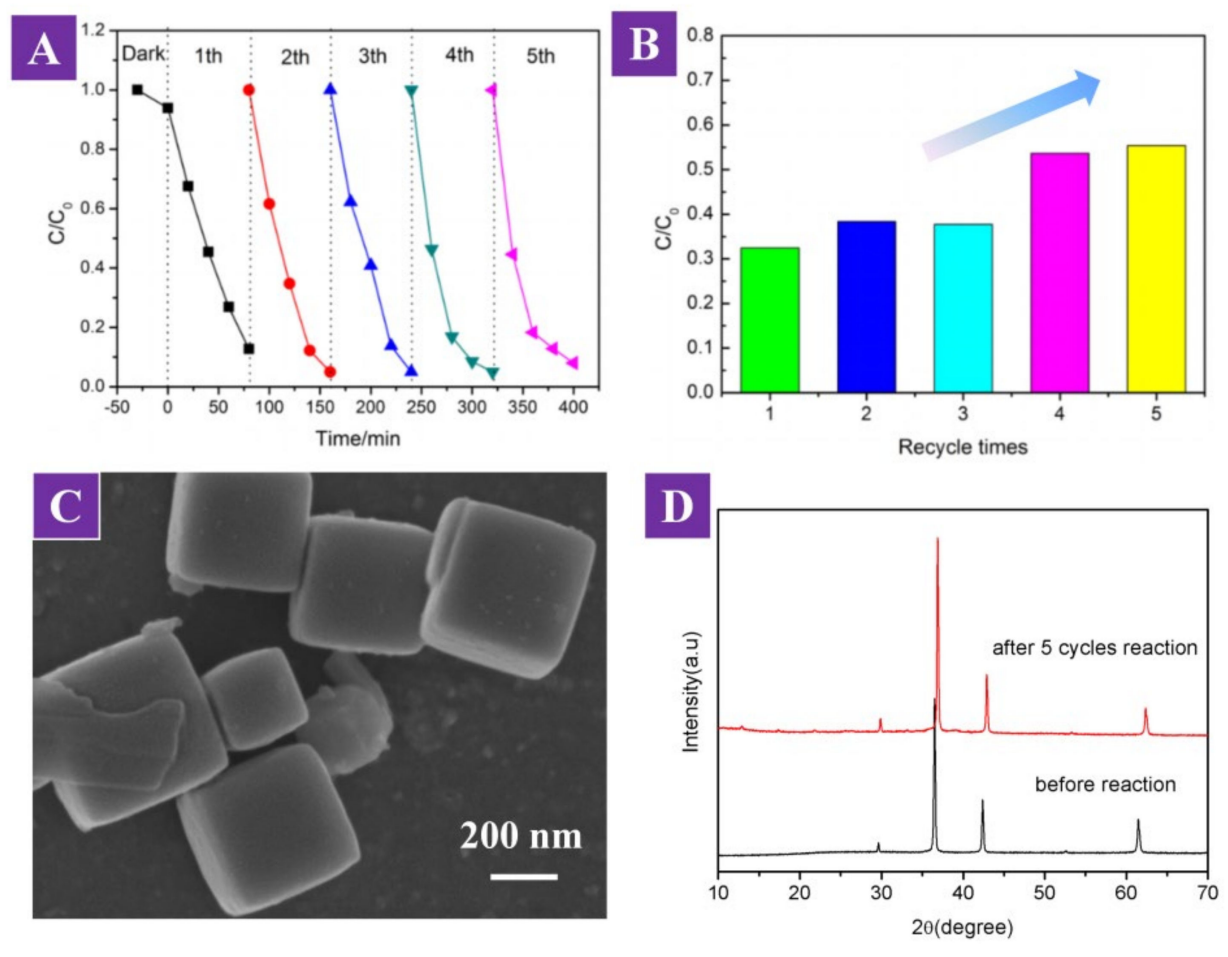
(**A**)The recycling experiments of c-Cu_2_O(100) nanocrystals toward the degradation of MO. (**B**) Comparison of the corresponding MO degradation after 20 min. (**C**) SEM images and (**D**) XRD of c-Cu_2_O(100) nanocrystals after five recycles.

Meanwhile, the MO photodegradation recycling experiments were carried out to investigate the stability of the c-Cu_2_O(100) nanocrystals. As shown in Figure 8A, the photocatalytic degradation rate nearly remained constant over five sequential cycles within 80 min under visible-light irradiation, indicating that c-Cu_2_O(100) nanocrystals possess a good stability during the photocatalytic reaction process. It is worth noting that the photodegradation rate increased gradually in the first 20 min for five cycles as shown in Figure 8B. According to the previous report [43], Cu_2_O is easily oxidized into CuO under illumination, except when adding a hole (h^+^) scavenger. Therefore, MO may act as a h^+^ scavenger, and facilitate rapid separation of electron and hole pairs, while leaving excess electrons, which may reduce CuO on the surface of c-Cu_2_O(100) to Cu_2_O. This process is beneficial for enhancing photocatalytic performance. SEM and XRD measurements were carried out to investigate catalysts after five cycles. As shown in Figure 8C, the shape of c-Cu_2_O(100) showed no change and remained in the cubic morphology after five cycles. The results of XRD showed that the crystal structure of c-Cu_2_O(100) after five cycles is consistent with that of pristine c-Cu_2_O(100) nanocrystals (Figure 8D). These results of SEM and XRD further demonstrate that c-Cu_2_O(100) nanocrystals possess a good stability.

### 3.7. Charge Transfer Dynamics and PEC Measurements

**Figure 9:**
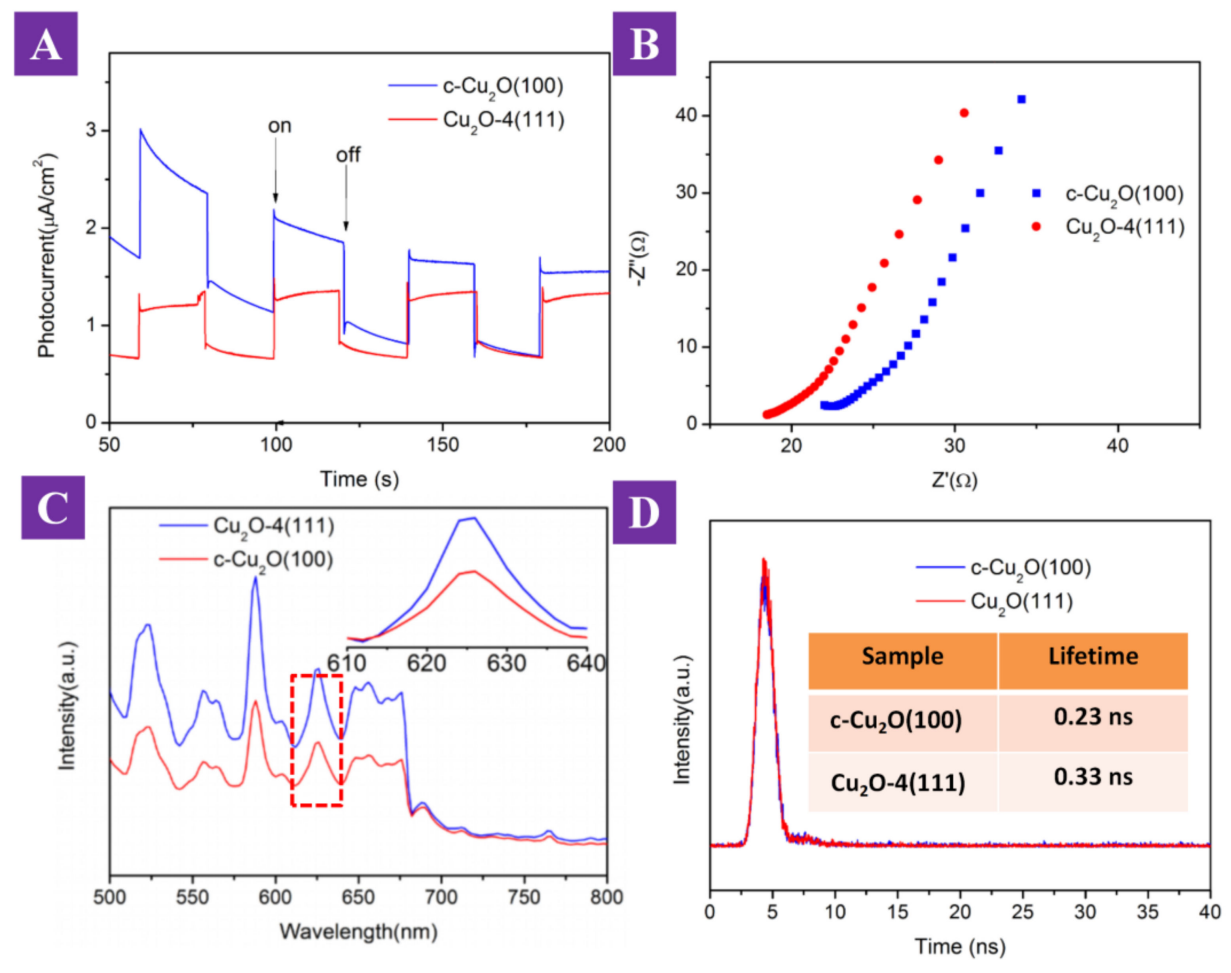
(**A**) Photocurrent response, (**B**) electrochemical impedance spectra, (**C**) PL spectra, and (**D**) TRPL decay curves of c-Cu_2_O(100) and Cu_2_O-4(111).

To gain deep insights into the separation efficiency of photogenerated electron–holes, PEC tests, PL and TRPL spectra performance were carried out [48]. Photocurrent responses and electrochemical impedance spectra are shown in Figure 9A,B, respectively. From these figures, it can be found that c-Cu_2_O (100) has a stronger photocurrent and weaker electrical resistance than Cu_2_O-4(111). This indicated that crystal surface (100) of Cu_2_O possessed higher electrical conductivity and carrier transmission speed than that of crystal surface (111). As shown in Figure 9C, c-Cu_2_O(100) displayed a lower PL intensity, which indicated a higher separation efficiency of the charge carriers than that in Cu_2_O-4(111). The TRPL spectra were further detected to understand the lifetime of photo-generated charge carriers as shown in Figure 9D. The lifetimes of c-Cu_2_O(100) and Cu_2_O-4(111) were 0.23 ns and 0.33 ns, respectively, indicating that the photo-generated carriers of c-Cu_2_O(100) were captured more rapidly by the reactive substrate and thus were able to drive the redox reactions [49]. Furthermore, c-Cu_2_O(100) had higher separation efficiency for electrons and holes.

### 3.8. Trapping Experiment of Cative Species

**Figure 10:**
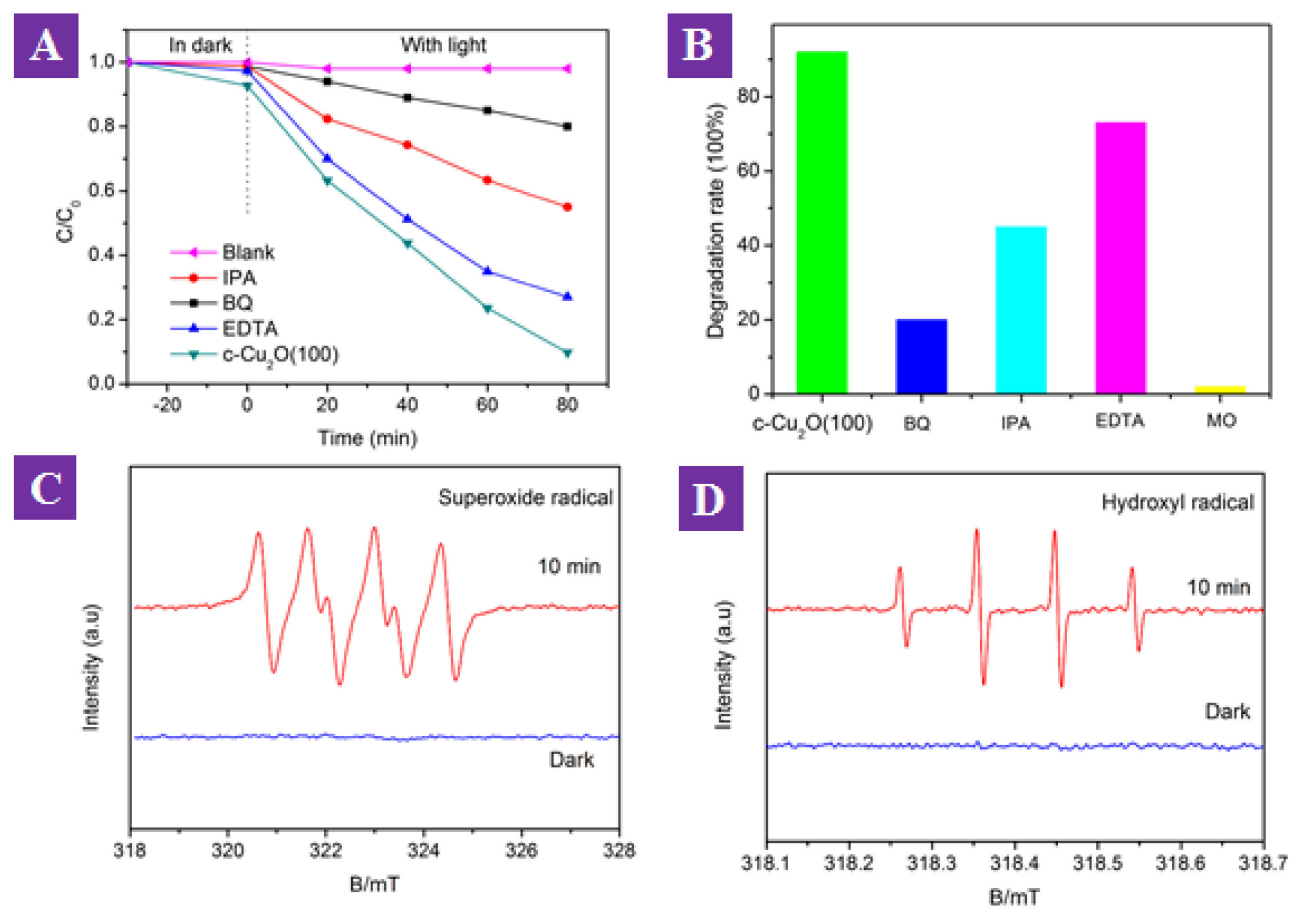
Photocatalytic degradation curve (**A**) and degradation rate (**B**) of MO using c-Cu_2_O nanocrystal photocatalyst with additions of scavengers under visible light, (**C**) ESR-DMPO−∙O_2_^−^ and (**D**) ESR-DMPO−·OH spectra of c-Cu_2_O nanocrystals.

In order to identify the main reactive species for the photocatalysis photodegradation MO process over Cu_2_O nanocrystals, active-species-trapping experiments were carried out. In detail, BQ, IPA and EDTA were introduced as scavengers for ∙O_2_^−^, ∙OH and holes [30,31,44,50], respectively. As shown in Figure 10A, the addition of EDTA slightly influenced the photocatalytic degradation rate of MO, which implied that few holes participated in the photocatalytic degradation reaction. Under the same condition, the addition of BQ and IPA obviously decreased the degradation rate of MO, which indicated that ∙O_2_^−^ and ∙OH are the key reactive species for MO oxidation. The corresponding MO degradation rate over c-Cu_2_O(100) with the addition of BQ, IPA and EDTA are 20.2%, 45.1% and 73.4% (Figure 10B). To further confirm the mechanism of enhanced photocatalytic degradation on Cu_2_O nanocrystals, the ESR was carried out to investigate the active radicals in solution. Spin capture reagent DMPO can be used to detect ∙OH and ∙O_2_^−^ in aqueous and methanol solution, respectively. DMPO can produce an ESR signal by reacting with the oxygen-containing group but it did not produce an ESR signal [51]. As shown in Figure 10C,D, no characteristic peaks of DMPO-∙O_2_^−^ and DMPO-∙OH can be found over the as-prepared Cu_2_O nanocrystals in the dark, which indicate that Cu_2_O nanocrystals cannot produce an ESR signal without irradiation. As shown in Figure 10C, after being given visible-light irradiation for 5 min, six characteristic peaks can be clearly observed, which belong to the characteristic signals of DMPO-∙O_2_^−^ [52]. With the increase in the light period length, the intensity of the ESR signal was significantly enhanced, which indicated that ∙O_2_^−^ radicals can be produced in the photocatalytic degradation reaction under visible-light irradiation. Similarly, four obvious characteristic peaks with the relative intensity of 1:2:2:1 can be observed after giving 5 min irradiation, which correspond to the characteristic signals of DMPO-∙OH. The signal intensity was obviously enhanced when increasing the irradiation time from 5 min to 10 min as shown in Figure 10D, which confirmed that the ∙OH radicals can be produced in the photocatalytic degradation under visible-light irradiation.

Based on the above results, it can be concluded that many charge carriers can be generated from the c-Cu_2_O nanocrystals after visible light irradiation. Subsequently, the electrons transferred to the surfaces of c-Cu_2_O nanocrystals and reacted with molecular oxygen in solution to form ∙O_2_^−^ radicals [52], and the holes transferred to the surfaces of c-Cu_2_O nanocrystals and oxidized the OH^−^ to ∙OH radicals [53]. Finally, active radicals participated in the redox reaction of dye MO. Thus, c-Cu_2_O nanocrystals with the efficient utilization of charge carriers displayed the excellent photocatalytic degradation performance.

### 3.9. Photocatalytic Mechanism

**Figure 11:**
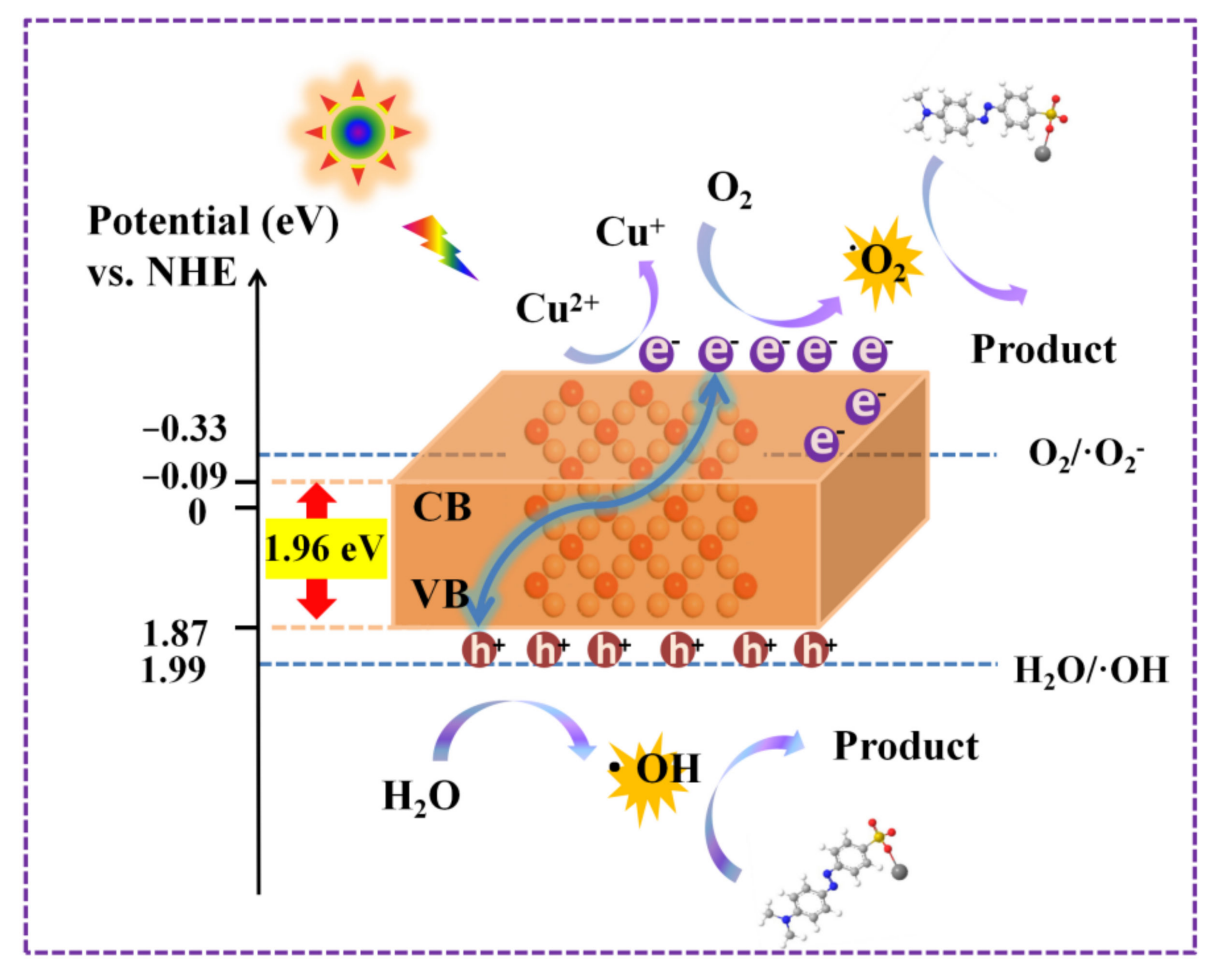
Schematic illustration of the MO degradation mechanism over Cu_2_O nanocrystals under visible light.

According to the previous reports and above results, the possible photocatalytic mechanisms are proposed. As shown in Figure 11, under visible-light irradiation, the Cu_2_O semiconductor is excited to generate e^−^-h^+^ pairs. e^−^ migrates to the CB and leaves h^+^ on the VB of Cu_2_O. e^−^ can react with molecular oxygen in solution to form ∙O_2_^−^ radicals (Equation (3)). Simultaneously, h^+^ reacts with OH^-^ in H_2_O to form ∙OH radicals (Equation (6)). However, the CB position of Cu_2_O (about −0.09 eV) is more positive than the redox potential of O_2_ to ∙O_2_^−^ (−0.33 eV) and the VB position of Cu_2_O (1.87 eV) is more negative than the redox potential of H_2_O to ∙OH (1.99 eV) [54]; these results did not correspond with those of ESR. This may be because the partial oxidation of the Cu_2_O surface generates CuO, which constitutes the heterojunction and thus affects the band gap position [55]. According the previous report [43], the reasons why Cu_2_O self-photooxidation does not happen in the absence of h^+^ scavenger may be that the MO dye acts as a h^+^ scavenger or generates CuO (Equation (8)) is reduced to Cu_2_O by accumulated photogenerated e^-^ in the CB of Cu_2_O (Equation (5)). Therefore, the redox pair of Cu^2+^-Cu^+^ has been formed and plays an important role in the photocatalytic reaction. For example, it enhances the separation and transfer of photogenerated charges. The circulation of the redox pair of Cu^2+^-Cu^+^ can generate more reactive species to improve the degradation performance [56]. Cu^+^ may be oxidized into Cu^2+^ by holes and Cu^2+^ may be reduced by electrons. The close coordination between “Cu^2+^-Cu^+^” redox pairs and electron–hole teams under light improves the stability of the catalytic system. Subsequently, ∙O_2_^−^, h^+^, and ∙OH radicals are the main active species to decompose MO dye to CO_2_ and H_2_O, and the possible reaction Equations (4), (7), and (9) are displayed, respectively.

CB: e^−^ + O_2_ →∙O_2_^−^ (3)

∙O_2_^−^ + Dye →CO_2_ + H_2_O (4)

Cu^2+^(CuO) + e^−^ → Cu^+^(Cu_2_O) (5)

VB: h^+^ + OH^−^ →∙OH (6)

h^+^ + Dye →CO_2_ + H_2_O (7)

h^+^ + Cu^+^(Cu_2_O) → Cu^2+^(CuO) (8)

∙OH + Dye →CO_2_ + H_2_O (9)

## 4. Conclusions

In summary, three shapes of Cu_2_O were first successfully obtained via a facile wet-chemical method at room temperature. This strategy is easy to operate and energy-efficient, and it will provide a new idea for the synthesis of Cu_2_O. Specifically, the morphologies of Cu_2_O change from cubic to spherical by simply adjusting the amounts of PVP at room temperature, and the dominant exposed surface of cubic Cu_2_O and spherical Cu_2_O are (100) and (111) surface, respectively. We investigated the relationship between structure and function by a photodegradation experiment. It was found that c-Cu_2_O(100) exhibited the best photocatalytic activity among the as-prepared samples. After optical properties’ characterization, PEC, PL and TRPL testing, it was found that c-Cu_2_O(100) exhibited higher efficiency of electron and hole separation than Cu_2_O-4(111), although they have similar light-absorbing properties. These results indicated that Cu_2_O exhibited crystal-plane-dependent properties and c-Cu_2_O(100) with exposing (100) facet possesses higher photocatalytic activity. Trapping experiments and ESR measurement showed that ∙O_2_^−^, h^+^, and ∙OH radicals participated in the photocatalytic process. In summary, the results and discussion will offer useful help for environmental purification and energy conversion over Cu_2_O-based photocatalysts.

## Data Availability

Data are contained within the article.

## Supplementary Materials

The following supporting information can be downloaded at: https://www.mdpi.com/article/10.3390/nano12101697/s1, Figure S1: Photocatalytic apparatus, Figure S2: TEM images of (A) c-Cu_2_O, (B) Cu_2_O-1 and (C) Cu_2_O-4, Figure S3: X-ray photoelectron spectroscopy (XPS) survey spectrum of c-Cu_2_O, Cu_2_O-1 and Cu_2_O-4, Figure S4: The O 1s XPS spectrum of (A) c-Cu2O, (B) Cu2O-1 and (C) Cu2O-4, Figure S5: (A) FTIR spectra of c- Cu_2_O, Cu_2_O-1 and Cu_2_O. (B) Corresponding to the enlarged views at 632 cm^−1^ and 1632 cm^−1^ in Figure A (marked by red dashed lines).
