# Supplementary material for: Room Temperature Engineering Crystal Facet of Cu2O for Photocatalytic Degradation of Methyl Orange
Source: Nanomaterials (Basel). 2022 May 16;12(10):1697. doi: 10.3390/nano12101697 (PMC9144586; doi:10.3390/nano12101697)
Supplement: Supplementary file 1 [file nanomaterials-12-01697-s001.zip › nanomaterials-1674122-supplementary.pdf]

nanomaterials

Supporting Materials

Room Temperature Engineering Crystal Facet of Cu_2O for Photocatalytic Degradation of Methyl Orange

Jiwen Li [1,2,*], Meizi He [1,3], Jiankun Yan [1], Jiahui Liu [1], Jiaxin Zhang [1,4] and Jingjun Ma [1,*]

1 College of Science and Technology, Hebei Agricultural University, Huanghua 061100, China; hemeizi21@mails.ucas.ac.cn (M.H.); jkyan007@foxmail.com (J.Y.); jiahuiliu97@163.com (J.L.); zhangjx0926@stumail.ysu.edu.cn (J.Z.)
2 Hefei National Research for Physical Sciences at the Microscale, CAS Center for Excellence in Nanoscience, University of Science and Technology of China, Hefei 230026, China
3 State Key Laboratory of Coal Conversion, Institute of Coal Chemistry, Chinese Academy of Sciences, Taiyuan 030001, China
4 College of Environmental and Chemical Engineering, Yanshan University, Qinhuangdao 066004, China
* Correspondence: lijiwen@hebau.edu.cn (J.L.); mjjwjpmartin@sina.com (J.M.); Tel./Fax: +86-317-560-5226 (J.M.)

Citation: Li, J.; He, M.; Yan, J.; Liu, J.; Zhang, J.; Ma, J. Room Temperature Engineering Crystal Facet of Cu_2O for Photocatalytic Degradation of Methyl Orange. *Nanomaterials* **2022**, *12*, 1697. https://doi.org/10.3390/nano12101697

Academic Editor: Younghun Kim

Received: 25 March 2022
Accepted: 9 May 2022
Published: 16 May 2022

Publisher's Note: MDPI stays neutral with regard to jurisdictional claims in published maps and institutional affiliations.

Copyright: © 2022 by the authors. Licensee MDPI, Basel, Switzerland. This article is an open access article distributed under the terms and conditions of the Creative Commons Attribution (CC BY) license (https://creativecommons.org/licenses/by/4.0/).

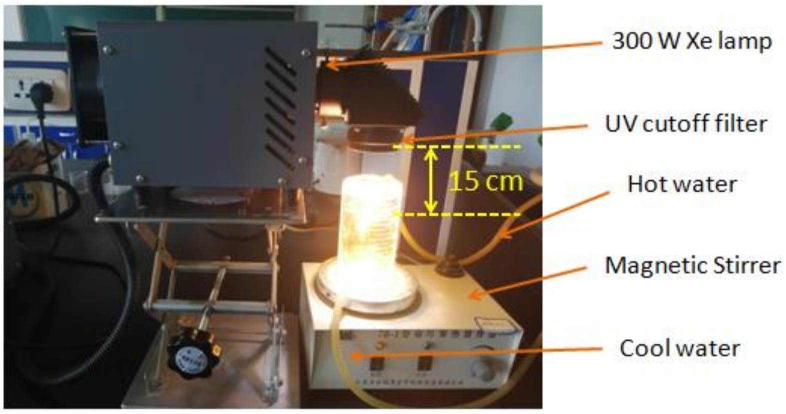

Figure S1. Photocatalytic apparatus.

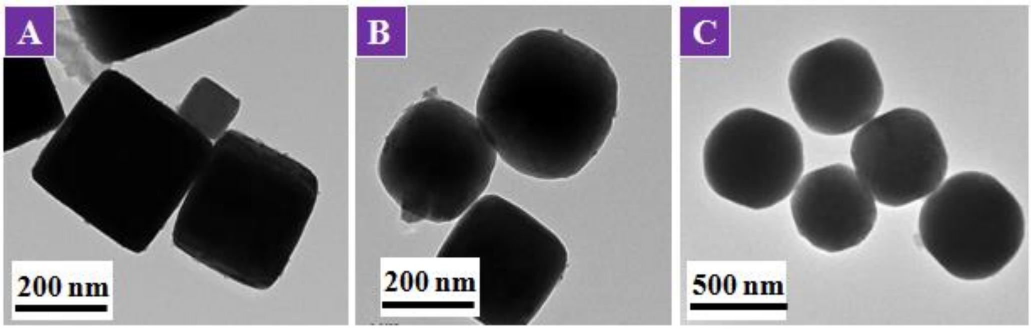

Figure S2. TEM images of (**A**) c-Cu_2O, (**B**) Cu_2O-1 and (**C**) Cu_2O-4.

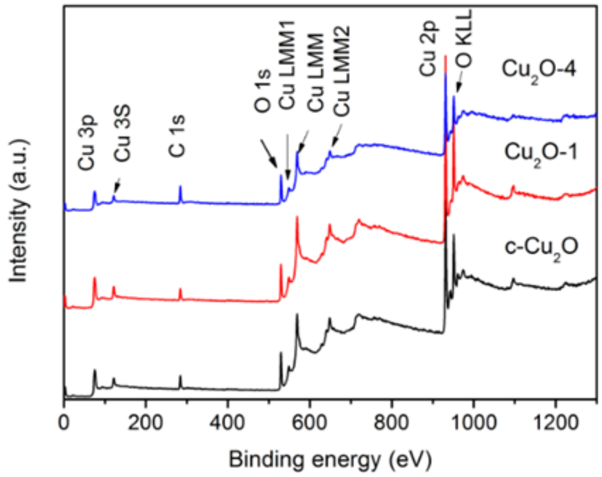

Figure S3. X-ray photoelectron spectroscopy (XPS) survey spectrum of c-Cu_2O, Cu_2O-1 and Cu_2O-4.

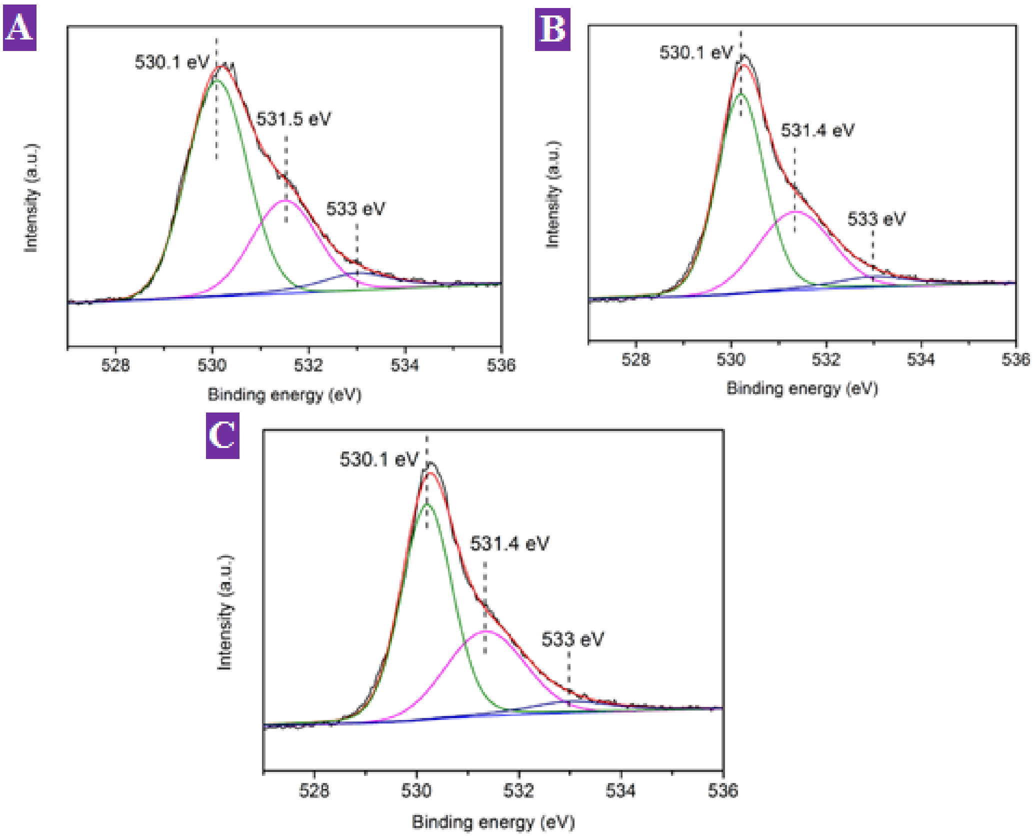

Figure S4. The O 1s XPS spectrum of (**A**) c-Cu_2O, (**B**) Cu_2O-1 and (**C**) Cu_2O-4.

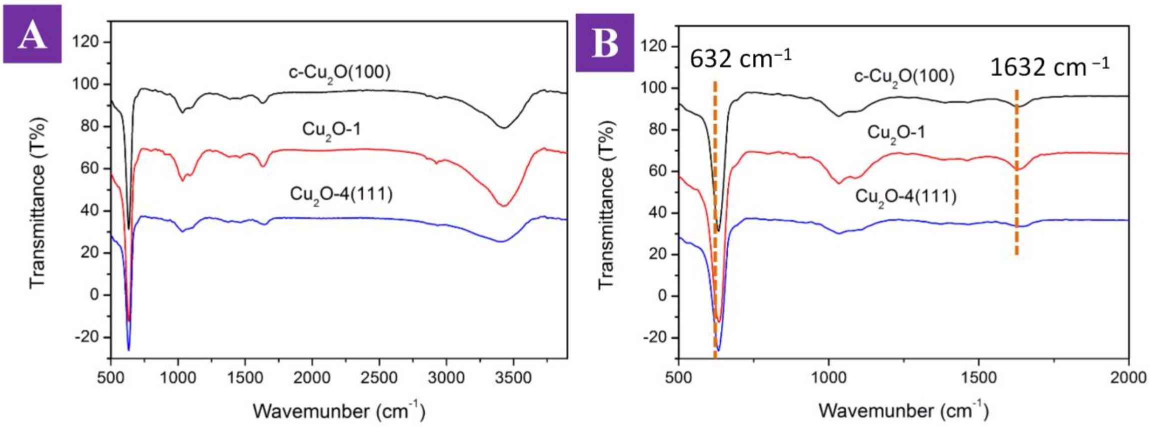

Figure S5. (**A**) FTIR spectra of c- Cu_2O, Cu_2O-1 and Cu_2O. (**B**) Corresponding to the enlarged views at 632 cm^{-1} and 1632 cm^{-1} in Figure A (marked by red dashed lines).
